# Does Timing of Radiation Therapy Impact Wound Healing in Patients Undergoing Metastatic Spine Surgery?

**DOI:** 10.3390/diagnostics14101059

**Published:** 2024-05-20

**Authors:** Ranbir Ahluwalia, Hani Chanbour, Tyler Zeoli, Amir M. Abtahi, Byron F. Stephens, Scott L. Zuckerman

**Affiliations:** 1 Department of Neurological Surgery, Vanderbilt University Medical Center, Medical Center North T-4224, Nashville, TN 37212, USAtyler.zeoli@vumc.org (T.Z.); 2 Department of Orthopedic Surgery, Vanderbilt University Medical Center, Nashville, TN 37232, USA

**Keywords:** spinal tumors, radiation, metastasis, wound complication, SBRT, EBRT, wound infection

## Abstract

Introduction: The impact of radiation on wound healing after metastatic spine surgery remains an active area of research. In patients undergoing metastatic spine surgery, we sought to (1) assess the relationship between preoperative and/or postoperative radiation on wound complications, and (2) evaluate the relationship between the timing of postoperative radiation and wound complications. Methods: A single-center, retrospective, cohort study of patients undergoing metastatic spine surgery was conducted from 2010 to 2021. The primary exposure variable was the use/timing of radiation. Radiation included both external beam radiotherapy (EBRT) and stereotactic body radiotherapy (SBRT). Patients were trichotomized into the following groups: (1) preoperative radiation only, (2) postoperative radiation only, and (3) no radiation. The primary outcome variable was wound complications, which was defined as dehiscence requiring reoperation, infection requiring antibiotics, or infection requiring surgical debridement. Multivariable logistic/linear regression controlled for age, tumor size, primary organ of origin, and the presence of other organ metastases. Results: A total of 207 patients underwent surgery for extradural spinal metastasis. Participants were divided into three groups: preoperative RT only (N = 29), postoperative RT only (N = 91), and no RT (N = 178). Patients who received postoperative RT only and no RT were significantly older than patients who received preoperative RT only (*p* = 0.009) and were less likely to be white (*p* < 0.001). No other significant differences were found in basic demographics, tumor characteristics, or intraoperative variables. Wound-related complications occurred in two (6.9%) patients with preoperative RT only, four patients (4.4%) in postoperative RT only, and 11 (6.2%) patients with no RT, with no significant difference among the three groups (*p* = 0.802). No significant difference was found in wound-related complications, reoperation, and time to wound complications between patients with preoperative RT only and no RT, and between postoperative RT only and no RT (*p* > 0.05). Among the postoperative-RT-only group, no difference in wound complications was seen between those receiving SBRT (5.6%) and EBRT (4.1%) (*p* > 0.999). However, patients who received preoperative RT only had a longer time to wound complications in comparison to those who received postoperative RT only (43.5 ± 6.3 vs. 19.7 ± 3.8, *p* = 0.004). Regarding timing of postoperative RT, the mean (SD) time to RT was 28.7 ± 10.0 days, with a median of 28.7 (21–38) days. No significant difference was found in time to postoperative RT between patients with and without wound complications (32.9 ± 12.3 vs. 29.0 ± 9.7 days, *p* = 0.391). Conclusion: In patients undergoing metastatic spine surgery, a history of previous RT or postoperative RT did not significantly affect wound complications. However, those with previous RT prior to surgery had a longer time to wound complications than patients undergoing postoperative RT only. Moreover, timing of RT had no impact on wound complications, indicating that earlier radiation may be safely employed to optimize tumor control without fear of compromising wound healing.

## 1. Introduction

The spinal column is the most common location for bony metastases in patients with cancer, with a rising incidence given the advances in treatment [1,2]. Up to 70% of cancer patients can develop spinal metastases [3], accounting for 18,000 new cases per year [4]. Metastatic disease of the spine often presents with pain, secondary to bony destruction, fracture, or epidural spinal cord compression [5,6]. Separation surgery, which includes the circumferential decompression of the spinal cord, reconstitution of the thecal sac, and stabilization, is the mainstay treatment, followed by postoperative stereotactic radiation shortly after surgery [7,8].

While postoperative RT offers the best means of local control, the ideal timing of radiation remains an active area of research. While some studies advocate for early radiation within 1–2 weeks [9,10], other studies recommend 4–6 weeks postoperative [11]**.** Moreover, the type of radiation is also an important question. The two major categories of spinal column radiation are the standard external beam radiation (EBRT) and the stereotactic body radiotherapy (SBRT); the first is the non-focused delivery of radiation that affects local tissue, compared to the newer SBRT, which delivers high-dose, focal radiation in small fractions. SBRT is best for radioresistant tumors, which are what most commonly require separation surgery [12]. When deciding when to start radiation, perhaps the most feared complication is wound dehiscence and/or infection [13]. The incidence of surgical site infections following spine surgery can range from 0.7 to 20% [14]. Comorbidities such as diabetes, malnutrition, smoking, and obesity have been associated with an increased chance of infection in patients with spinal tumors [15]. The few studies that have evaluated the timing of postoperative radiation in metastatic spine surgery have found no significant differences in wound healing when comparing early vs. delayed radiation [10,16].

The importance of early postoperative radiation to achieve local control along with the overall goal of avoiding wound complications are two areas in need of further reconciliation [17]. Few studies have evaluated the effects of radiation therapy on wound healing in patients with metastatic spine disease [18,19]. The objectives of the current study were to (1) assess the relationship between preoperative and/or postoperative radiation on wound complications, and (2) evaluate the relationship between timing of postoperative radiation and wound complications.

## 2. Methods

### 2.1. Study Design

A single-institution, multi-surgeon, case–control study was performed for patients undergoing spinal tumor surgery from 2010 to 2021. The institutional review board (IRB) approach was obtained for this study (IRB#211900). Consent was not required due to the retrospective nature of the study.

### 2.2. Patient Population

Registry data were collected and analyzed for patients who underwent surgery for spinal metastasis. Inclusion criteria included patients who were at least 18 years of age with metastatic extradural spinal tumors ultimately requiring tumor resection and/or stabilization. Exclusion criteria included pediatric patients (less than 18 years of age), intradural tumors, primary tumors, and those without preoperative T2-weighted MRIs. Follow-up time was extended to date of death or last clinical follow-up.

### 2.3. Exposure Variable

The primary exposure variable was the use/timing of radiation. Radiation included both EBRT and SBRT, as well as preoperative and postoperative radiation. Patients were divided into the following 3 groups: (1) preoperative radiation only, (2) postoperative radiation only, (3) no radiation at all. Given the a priori goal of the study, patients who received RT after 6 weeks were excluded, so we could evaluate the true impact of radiation on wound healing, as most wounds are healed by 6 weeks. The 6-week number was chosen a priori based on maximum time a wound can heal [20].

Additional exposure variables included demographics, cancer-specific variables, and perioperative variables. Preoperative variables included age, BMI, comorbidities, tumor’s primary organ, tumor size (levels), the number of metastases specific to the spinal column, smoking status, other organ metastases, and time to last follow-up. Perioperative variables included functional and pain status at presentation, and the use of preoperative embolization. Intraoperative variables included surgical procedure, total instrumented levels, total decompressed levels, estimated blood loss, and operative time. Postoperative complications, length of stay (LOS), and discharge disposition were also recorded.

### 2.4. Outcome Variable

The primary outcome was wound complications, which was defined as dehiscence requiring reoperation, infection requiring antibiotics, or infection requiring surgical debridement. Amongst all three groups, mean differences were evaluated for time to wound complication. Similarly, mean differences were evaluated for wound complication and reoperation rate. Wound-related complications, wound reoperation, and time to wound complication was then evaluated using both univariate/multivariable linear/logistic regression controlling for age, tumor size, primary organ of origin, and the presence of other organ metastases.

### 2.5. Surgical Treatment

All patients underwent separation surgery, including the decompression of the spinal cord with long-segment posterior stabilization and fusion [21]. In order to achieve spinal cord decompression, posterior approach was often supplemented with a transpedicular decompression or costotransversectomy. The ultimate goal was to achieve separation between the tumor and spinal cord with the reconstitution of the thecal sac. Ultrasound can be used as an adjunct therapy to ensure adequate decompression. As such, a safe distance (2–3 mm) was created between the tumor and thecal sac to minimize collateral damage from radiation treatment. Stabilization included standard pedicle screw/lateral mass fixation two to three levels above and below the site of decompression. Anterior column reconstruction was not routinely performed, but rather, only in cases of a lytic lesion or determined on a case-by-case basis by the surgeon. Postoperative radiotherapy consisted of either EBRT and SBRT. Radiotherapy type was mutually decided between the spinal surgeon and radiation oncologist. 

### 2.6. Statistical Analysis

Descriptive statistics were reported to compare patients who did and did not receive preoperative radiation therapy. Mean and standard deviation were reported for continuous variables and frequency for categorical variables. Normal distribution and variance for continuous variables were assessed with the Shapiro–Wilk test and F test, respectively. When comparing three groups, ANOVA was used for continuous variables and chi-squared was used for categorical variables. For nonparametric data, the Kruskal—Wallis and chi-squared tests were used for continuous and categorical data, respectively. For the outcome variable comparing two groups, parametric data with equal variance were analyzed with a two-tailed *t*-test, while nonparametric data were compared with the Wilcoxon signed-rank or Mann–Whitney test. Univariate/multivariable logistic/linear regressions were performed, controlling for age, tumor size, primary organ of origin, and the presence of other organ metastases. α value < 0.05 was considered statistically significant. All analyses were performed using IBM SPSS Statistics 25.0.

## 3. Results

### 3.1. Preoperative and Perioperative Data

A total of 207 patients underwent surgery for extradural spinal metastasis. Groups were divided into the following: (1) preoperative RT only (N = 29), (2) postoperative RT only (N = 91), and (3) no RT (N = 178). 

Comparing demographics, patients who received postoperative RT only and no RT were significantly older than patients who received preoperative RT only (*p* = 0.009) and were less likely to be white (*p* < 0.001). No other significant differences were found in basic demographics, the presence of other organ metastases (*p* = 0.061), the primary organ of origin (*p* = 0.348), motor deficit (*p* = 0.848), preoperative Karnofsky performance scale (KPS) (*p* = 0.559), tumor location (*p* = 0.178), and tumor size (*p* = 0.190) (Table 1).

Perioperatively, no significant differences were found in the type of surgery performed, operative time (*p* = 0.560), EBL (*p* = 0.874), length of stay (*p* = 0.638), postoperative disposition (*p* = 0.624), or discharge home (*p* = 0.361) (Table 2).

### 3.2. Wound Complications

Wound-related complications occurred in a total of 17 (5.7%) patients, with the following breakdown: preoperative RT only, two patients (6.9%); postoperative RT only, four patients (4.4%); and patients with no RT, 11 patients (6.2%), with no significant difference among the three groups (*p* = 0.802). Similarly, no significant differences were found in wound reoperation (*p* = 0.918) and time to wound complication (*p* = 0.519) among the three groups (Table 2).

When comparing two groups against each other, no significant difference was found in wound-related complications, reoperation, and time to wound complications between patients with preoperative RT only and no RT, postoperative RT only and no RT, and postoperative SBRT vs. postoperative non-SBRT. However, patients who received preoperative RT only had a longer time to wound complications in comparison to those who received postoperative RT only (43.5 ± 6.3 vs. 19.7 ± 3.8, *p* = 0.0004) (Table 3). While this association was statistically significant on univariate logistic regression (OR = 23.7, 95%CI = 12.65–34.85, *p* = 0.004), only an insufficient sample size was available for a multivariable analysis. Multivariable regression analysis controlling for age, tumor size, primary organ of origin, and the presence of other organ metastases predicting wound complication is presented in Table 4.

All patients who had wound complications are described in Table 5. Of note, although these patients presented with a different tumor histology, we have incorporated the primary organ of origin in the multivariable regression as a controlling factor, and there was no significant difference in tumor histology at baseline between the three groups. Of the patients who received preoperative RT only, two had wound-related complications, and only one of them required reoperation. The mean time to wound complication was 43.5 ± 6.3 days. Of the patients who underwent postoperative radiation within 6 weeks, four endured a wound-related complication, while all of them required reoperation. The mean time to wound complication was 19.7 ± 3.8 days. Of the patients who received no postoperative RT, eleven endured a wound complication, nine required reoperation, and the mean time to wound complication was 44.0 ± 42.7 days. Of the patients who received postoperative SBRT, one patient endured a wound complication and required reoperation, and 21 days elapsed until the complication occurred. Of the patients who received postoperative EBRT, three endured a wound-related complication, and all patients required reoperation with a mean time of 19.3 ± 4.6 days. 

### 3.3. Radiation Timing

The mean (SD) time to RT was 28.7 ± 10.0 days, with a median of 28.7 (21–38) days. Grouped into categories, nine (9.9%) were started within 2 weeks, 39 (42.9%) within 2–4 weeks, and 43 (47.2%) within 4–6 weeks. 

**Figure 1:**
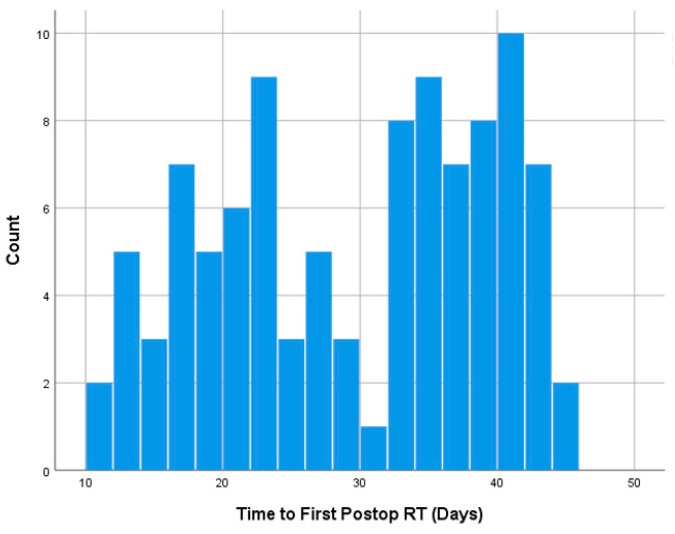
Histogram showing the distribution of time to first postop RT.

**Figure 2:**
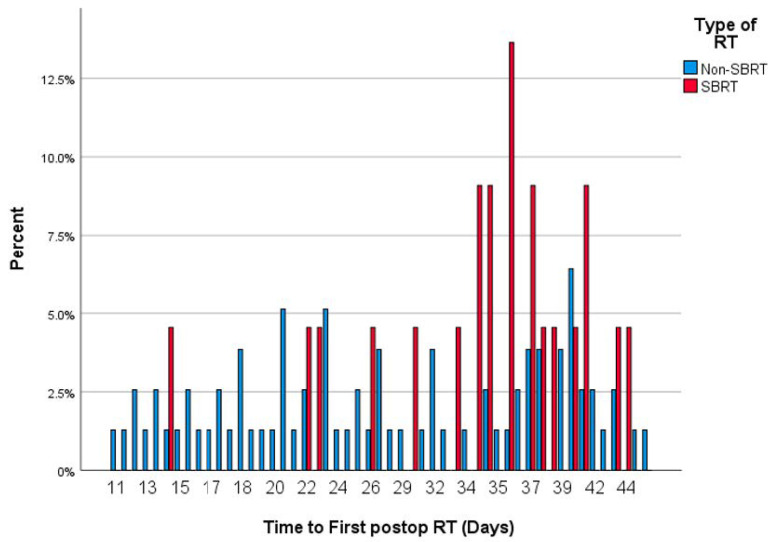
Histogram showing the distribution of time to first SBRT or EBRT.

**Figure 3:**
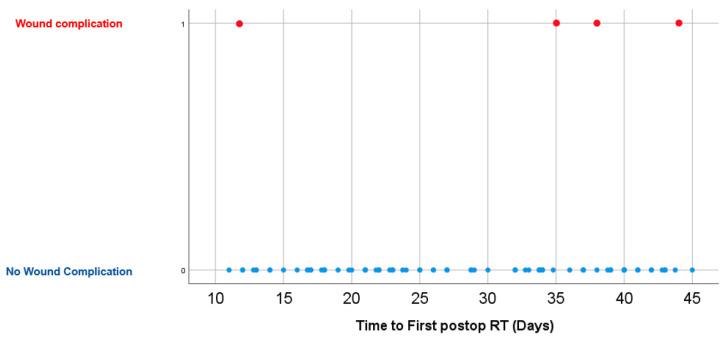
Scatter plot of time to wound complication and time to first postoperative RT.

No significant difference was found in time to postoperative RT between patients with and without wound complications (32.9 ± 12.3 vs. 29.0 ± 9.7 days, *p* = 0.391). Similar non-significant findings were observed on univariate (OR = 1.04, 95%CI = 0.93–1.16, *p* = 0.478) and multivariable logistic regression analyses (OR = 1.04, 95%CI = 0.94–1.15, *p* = 0.395) controlling for age, tumor size, primary organ of origin, and the presence of other organ metastases. Time to radiation had a bimodal distribution centered around 30 days. When stratifying between SBRT and non-SBRT, there was a rightward skew for SBRT patients. The distribution of time to postop RT is illustrated in Figure 1, Figure 2 and Figure 3.

**Figure 4:**
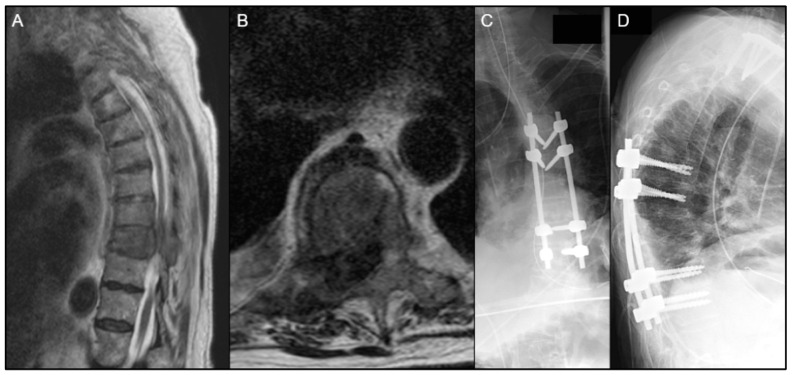
(**A**–**D**) A 78-year-old female with a history of stage IV metastatic breast cancer presented with urinary retention and lower extremity numbness within a 4-day duration, who was found to have metastatic tumor causing spinal cord compression with pathologic fracture at T8–T9 on T2-weighted MRI (**A**,**B**). She underwent separation surgery with T6–T11 posterior spinal instrumentation and fusion; T8, T9, and T10 laminectomy; right-sided T9 transpedicular decompression; and inferior facetectomies from T6 to T11, as seen on postoperative posteroanterior (**C**) and lateral (**D**) X-rays. Intraoperative ultrasound and neuromonitoring were used. The patient received postoperative EBRT after 1 month, and had wound infection 10 days after radiation, which required surgical debridement.

**Figure 5:**
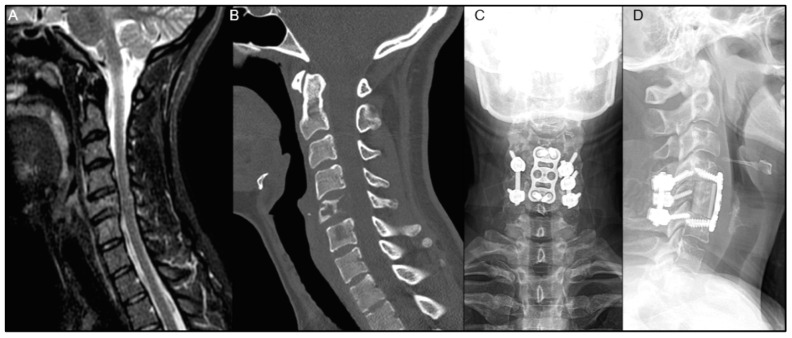
(**A**–**D**) A 26-year-old female presented with severe mechanical neck pain for several months and was found to have a C5 lytic lesion affecting 70% of the vertebral body as seen on preoperative sagittal T2-weighted MRI (**A**) and CT scan (**B**). The patient also had a left C6 radiculopathy, along with numbness and paresthesia, but with no reported weakness. She underwent separation surgery with a C5 corpectomy and C4–C6 anterior fusion with fibular allograft, as well as a posterior C4–C6 instrumented fusion, as seen on postoperative posteroanterior (**C**) and lateral (**D**) X-rays. The patient received SBRT for 2 weeks postoperatively and did not encounter any postoperative wound complications.

An illustrative case is described in Figure 4A–D of a patient who received postoperative EBRT after 1 month postoperatively, and had wound infection 10 days after RT, which required surgical debridement. Although no clear distinction was made regarding the exact etiology of the postoperative infection, radiation might have played a role in hindering the wound healing process. Figure 5A–D describe a patient who received SBRT 2 weeks postoperatively and did not encounter any postoperative wound complications.

## 4. Discussion

The purpose of this study was to determine the impact of preoperative, postoperative, and no radiation therapy on wound healing in patients undergoing metastatic spine tumor surgery. No difference was seen in wound complications comparing the three groups of preoperative radiation, postoperative radiation, and no radiation. The only significant difference found was that those with preoperative radiation only had a longer time to wound complication than those receiving postoperative radiation. Moreover, timing of radiation had no impact on wound complications either. These data suggest relative safety in early radiation to maximize tumor control. 

There were no significant differences in the rate of wound complications between patients who received preoperative radiation therapy, and those who did not receive any radiation. Vargas et al. [18] similarly found no difference in wound complication rate between patients who received preoperative radiation and those who did not. Competing risk analysis demonstrated a higher cumulative incidence of wound complications for patients with preoperative radiation, although this did not reach statistical significance in their study [18]. The overall wound complication rate was 14.3% for those who received preoperative radiation, and 11.5% for those who did not. This is in comparison to the current study which demonstrated a wound complication rate of 6.9% (preoperative radiation) and 6.2% (no radiation). Taken together, these findings suggest that the presence of preoperative radiation may have minimal impact on wound complications in comparison to those who are radiation naïve. To our knowledge, no other study directly compares wound complication rates in those who received preoperative radiation to those who are radiation naïve. 

In patients who received postoperative radiation within 6 weeks, there was a shorter duration to wound complication in comparison to patients who only received preoperative radiation therapy. Ghogawala et al. [19] performed a similar comparison in a cohort of 123 patients either undergoing radiation only, radiation followed by surgery, or de novo surgery followed by radiation. Interestingly, patients who had radiation prior to surgery had a threefold higher rate of wound complication compared to those who had de novo surgery followed by radiation. While the current study detected no change in the overall rate of complication between the same groups, the time to wound complication was shorter for patients receiving postoperative radiation. However, both Ghogawala’s study [19] and the present study are subject to limited sample sizes. Potential reasons for our findings include soft tissue fibrosis, epithelial ulceration, and fistula formation [22]. Furthermore, the current recommendation is one to five fractions delivering 5–24 Gy per fraction [23], which was adopted at our institution for all patients. A higher dose of radiation may be linked to skin changes, fatigue, radiation induced myelopathy, dysphagia/dysphonia (cervical spine), and GI symptoms (nausea, vomiting, diarrhea, or abdominal cramps) in the thoracic and lumbar spine, among others [23,24].

When comparing patients who have just received postoperative radiation (SBRT vs. non-SBRT), there was no difference in wound-related complications, wound reoperation rate, or time to wound complications. Similarly, Keam et al. [25] found no association between conventional fractionated radiotherapy or high-dose hypofractionated image-guided radiation therapy (IGRT) for spinal metastasis as predictors of wound complication in a cohort of 165 patients. Theoretically, SBRT should have a lower impact on wound healing due to the focal dose of radiation, but the current study may have been underpowered to detect a difference.

Ultimately, there is still a large amount of uncertainty on determining exactly when a patient should undergo preoperative and postoperative radiation. Azad et al. [16] identified a cohort of 540 patients undergoing early (within 4 weeks) vs. late (4 to 8 weeks) treatment and determined that there were no significant differences related to wound complications. When querying leading radiation oncologists and spine surgeons, there continues to be heterogeneity in answering this question. A general consensus is that there should be a one- to two-week gap after preoperative radiation before spine surgery, and that radiation can continue one to two weeks postoperatively [9]. The most comprehensive systematic review on this topic completed by Itshayek et al. [10] including 51 reports and 7090 patients concluded that waiting one week before and after surgery is a “safe window” for radiation. The present study seems to indicate that the group at highest risk for wound complication receive postoperative radiation within 6 weeks. However, even in this group, the rate of complication is exceedingly low. This finding, coupled with the prior literature, allows for a more balanced discussion with patients when identifying the unique risks of an operation. 

There are numerous limitations to this study. First, this was a retrospective cohort study and therefore has intrinsic limitations such as selection bias [26]. Second, the sample size was limited in the present study, and care should be taken not to overgeneralize the results. Third, wound healing is a complex process of which radiation is just an aspect. It would be impossible to control for all factors including postoperative resources, genetic factors, patient hygiene, suture material, etc. Therefore, several confounders of wound healing that were not analyzed in this study must exist. Fourth, time to preoperative radiation has a wide range and the effects of recent vs. distant preoperative radiation were not evaluated. Fifth, the senior author felt that six weeks was a reasonable cutoff for postoperative radiation. However, there is no consensus in the literature as to what defines early vs. late postoperative radiation. Finally, although this was a single-center study, there are differences in operative and therefore closure techniques amongst surgeons. 

## 5. Conclusions

The ideal timing of radiation after metastatic spine surgery continues to remain debated in the literature. No difference was seen in wound complications in our three groups of preoperative radiation only, postoperative radiation only, and no radiation. Interestingly, faster time to wound complications was seen in patients undergoing postoperative radiation compared to preoperative radiation. Moreover, timing of postoperative radiation had no impact on wound complications. These findings suggest that early radiation may not only optimize tumor control but also pose relatively low risk from a wound healing standpoint.

## Figures and Tables

**Table 1:** Demographics and preoperative variables.

	Preop RT Only N = 29	Postop RT Only N = 91	No RT N = 178	*p*-Value
Age mean ± SD	54.3 ± 15.6	61.7 ± 10.0	61.5 ± 12.2	0.009
BMI mean ± SD	26.8 ± 6.6	27.5 ± 6.7	27.2 ± 7.2	0.912
Gender, male n (%)	17 (58.6%)	54 (59.3%)	112 (62.9%)	0.806
Race, white n (%)	22 (75.9%)	82 (90.1%)	156 (87.6%)	<0.001
Other organ metastases, n (%)	20 (69.0%)	51 (56.0%)	84 (47.2%)	0.061
Primary organ, n (%)				0.348
Breast	2 (6.9%)	12 (13.2%)	15 (8.4%)	
Lung	4 (13.8%)	22 (24.2%)	47 (26.4%)	
Renal	4 (13.8%)	14 (15.4%)	17 (9.6%)	
Others	19 (65.5%)	43 (47.3%)	99 (55.6%)	
Time to last follow-up, mean ± SD	342.8 ± 239.3	436.4 ± 482.4	551.9 ± 720.0	0.337
Motor deficit, n (%)	13 (44.8%)	44 (48.4%)	89 (50.0%)	0.848
Preop KPS, mean ± SD	68.1 ± 15.4	64.6 ± 16.07	64.4 ± 17.7	0.559
Tumor locations, n (%)				0.178
Cervical	3 (10.3%)	12 (13.2%)	26 (14.6%)	
Cervicothoracic	3 (10.3%)	1 (1.1%)	6 (3.4%)	
Thoracolumbar	17 (10.3%)	61 (67.0%)	99 (55.6%)	
lumbar	6 (20.7%)	17 (18.7%)	47 (26.4%)	
Tumor size, mean ± SD	2.1 ± 1.7	1.8 ± 1.6	1.6 ± 1.2	0.190

**Table 2:** Intraoperative and postoperative variables.

	Preop RT Only N = 29	Postop RT Only N = 91	No RT N = 178	*p*-Value
Instrumented, n (%)	27 (93.1%)	90 (98.9%)	173 (97.2%)	0.199
Decompressed, n (%)	26 (89.7%)	86 (94.5%)	170 (95.5%)	0.337
Total decompressed levels, mean ± SD	2.4 ± 1.5	2.8 ± 1.4	2.5 ± 1.36	0.253
Total instrumented levels, mean ± SD	5.5 ± 2.3	5.4 ± 2.1	5.5 ± 2.3	0.912
Transpedicular decompression, n (%)	13 (44.8%)	58 (63.7%)	86 (48.3%)	0.038
Costotransversectomy, n (%)	2 (6.9%)	16 (17.6%)	20 (11.2%)	0.205
Corpectomy/vertebrectomy, n (%)	11 (37.9%)	54 (59.3%)	96 (53.9%)	0.131
Operative time, mean ± SD	315.8 ± 119.9	297.5 ± 91.0	312.7 ± 126.6	0.560
EBL (mL), mean ± SD	809.1 ± 794.0	869.3 ± 1044.5	900.2 ± 864.6	0.874
LOS (days), mean ± SD	5.9 ± 4.5	7.0 ± 6.4	7.0 ± 5.8	0.638
Postop disposition, n (%)				0.624
Floor	17 (58.6%)	51 (56.0%)	91 (51.1%)	
ICU	12 (41.4%)	40 (44.0%)	87 (48.9%)	
Discharge home, n (%)	22 (75.9%)	48 (52.7%)	99 (55.6%)	0.361
Wound-related complications, n (%)	2 (6.9%)	4 (4.4%)	11 (6.2%)	0.802
Wound reoperation, n (%)	1 (3.4%)	4 (4.4%)	9 (5.1%)	0.918
Time to wound complications, mean ± SD	43.5 ± 6.3	19.7 ± 3.8	44.0 ± 42.7	0.519

**Table 3:** Wound complication and reoperation rate.

	Preop RT only N = 29	No RT N = 178	*p*-value
Wound-related complications, n (%)	2 (6.9%)	11 (6.2%)	>0.999
Wound reoperation, n (%)	1 (3.4%)	9 (5.1%)	>0.999
Time to wound complications, mean ± SD	43.5 ± 6.3	44.0 ± 42.7	0.985
	Preop RT only N = 29	Postop RT only (6W) N = 91	*p*-value
Wound-related complications, n (%)	2 (6.9%)	4 (4.4%)	0.631
Wound reoperation, n (%)	1 (3.4%)	4 (4.4%)	>0.999
Time to wound complications, mean ± SD	43.5 ± 6.3	19.7 ± 3.8	0.004
	Postop RT only (6W) N = 91	No RT N = 178	*p*-value
Wound-related complications, n (%)	4 (4.4%)	11 (6.2%)	0.780
Wound reoperation, n (%)	4 (4.4%)	9 (5.1%)	>0.999
Time to wound complications, mean ± SD	19.7 ± 3.8	44.0 ± 42.7	0.286
	Postop SBRT (6W) N = 18	Postop EBRT (6W) N = 73	*p*-value
Wound-related complications, n (%)	1 (5.6%)	3 (4.1%)	>0.999
Wound reoperation, n (%)	1 (5.6%)	3 (4.1%)	>0.999
Time to wound complications, mean ± SD	21.0	19.3 ± 4.6	0.784

**Table 4:** Linear/logistic regression, controlling for age, tumor size, primary organ of origin, and the presence of other organ metastases.

		Univariate		Multivariable	
Independent Variable	Outcome	β/OR (95%CI)	*p*-Value	β/OR (95%CI)	*p*-Value
Preop RT only vs. no RT	Wound-related complications	1.12 (0.23–5.35)	0.883	1.26 (0.24–6.65)	0.779
	Wound reoperation	0.67 (0.08–5.50)	0.710	0.81 (0.09–7.37)	0.858
	Time to wound complications	−0.59 (−69.5, 69.3)	0.985	6.94 (−91.59, 105.5)	0.872
Preop RT only vs. postop RT (6W)	Wound-related complications	1.61 (0.28–9.28)	0.594	2.58 (0.33–19.63)	0.360
	Wound reoperation	0.77 (0.08–7.24)	0.824	1.12 (0.09–13.94)	0.926
	Time to wound complications	23.7 (12.65–34.85)	0.004	-	**-**
Postop RT (6W) vs. no RT	Wound-related complications	0.69 (0.21–2.25)	0.548	0.70 (0.21–2.29)	0.560
	Wound reoperation	0.86 (0.25–2.88)	0.811	0.86 (0.25–2.90)	0.811
	Time to wound complications	−24.34 (−71.64, 22.96)	0.286	−42.40 (−124.61, 39.81)	0.273

**Table 5:** A detailed description of all wound complication cases. M: male, F: female, RCC: renal-cell carcinoma, ADK: adenocarcinoma, RT: radiation, EBRT: external beam RT, SBRT: stereotactic body RT, ENT: ear, nose, throat.

Patient	Age/Sex	Primary Organ	Instrumented Levels	Radiation	Time to Wound Complication	Reoperation for Wound Complication
1	58M	RCC	T10-L2	Preop RT only	48 days	No
2	69M	Prostate ADK	T2-T9	Preop RT only	39 days	Yes
3	44M	Lung ADK	T5-T9	Postop EBRT	14 days	Yes
4	56F	Lung ADK	O-C5	Postop EBRT	22 days	Yes
5	72M	Lung ADK	T1-T6	Postop SBRT	21 days	Yes
6	52M	Lung ADK	C5-T5	Postop EBRT	22 days	Yes
7	55M	Lung neuroendocrine	T5-T10	No RT	43 days	Yes
8	67F	Unknown carcinoma	T11-S1	No RT	155 days	No
9	74F	Lung neuroendocrine	T11-L3	No RT	22 days	Yes
10	68F	Leiomyosarcoma	L3-S1	No RT	23 days	Yes
11	75M	Adenocarcinoma	L1-L3	No RT	45 days	Yes
12	65F	Thyroid	L4-S1	No RT	20 days	Yes
13	68M	Lung ADK	L1-L3	No RT	84 days	Yes
14	63M	Squamous-cell carcinoma/ENT	C7-T9	No RT	32 days	Yes
15	65M	Melanoma	L1-L4	No RT	7 days	No
16	56M	Squamous-cell carcinoma/ENT	T8-T10	No RT	8 days	Yes
17	56F	RCC	T7-T11	No RT	46 days	Yes

## Data Availability

Data supporting the results of this paper are stored within Vanderbilt private databases and no results were formed using public databases.
